# The role of mitochondria in yeast programmed cell death

**DOI:** 10.3389/fonc.2012.00070

**Published:** 2012-07-03

**Authors:** Nicoletta Guaragnella, Maša Ždralević, Lucia Antonacci, Salvatore Passarella, Ersilia Marra, Sergio Giannattasio

**Affiliations:** ^1^ Institute of Biomembranes and Bioenergetics, National Research Council of Italy,Bari, Italy; ^2^ Department of Medicine and Health Sciences, University of Molise,Campobasso, Italy

**Keywords:** yeast, programmed cell death, mitochondria, acetic acid, cytochrome *c*, protein trafficking, intracellular signaling

## Abstract

Mammalian apoptosis and yeast programmed cell death (PCD) share a variety of features including reactive oxygen species production, protease activity and a major role played by mitochondria. In view of this, and of the distinctive characteristics differentiating yeast and multicellular organism PCD, the mitochondrial contribution to cell death in the genetically tractable yeast *Saccharomyces cerevisiae* has been intensively investigated. In this mini-review we report whether and how yeast mitochondrial function and proteins belonging to oxidative phosphorylation, protein trafficking into and out of mitochondria, and mitochondrial dynamics, play a role in PCD. Since in PCD many processes take place over time, emphasis will be placed on an experimental model based on acetic acid-induced PCD (AA-PCD) which has the unique feature of having been investigated as a function of time. As will be described there are at least two AA-PCD pathways each with a multifaceted role played by mitochondrial components, in particular by cytochrome *c*.

The unicellular yeast *Saccharomyces cerevisiae* has been established as a good model to elucidate molecular mechanisms underlying programmed cell death (PCD) pathways. *S. cerevisiae* PCD shares many morphological and biochemical features with apoptosis, the major form of mammalian PCD, although there are some peculiar differences. PCD have been described to occur in yeast in different physiological scenarios (Carmona-Gutierrez et al., 2010). Indeed, chromatin condensation, nuclear DNA fragmentation and phosphatidylserine externalization onto the cell surface are general markers of both mammalian and yeast PCD cells. A characteristic feature of mammalian apoptosis is the activation of caspases, proteases that initiate and execute cell death through degradation of cell components. Yeast contains only one gene homolog of caspases, named *YCA1*, encoding for yeast metacaspase (Madeo et al., 2002) which has substrate specificity different from caspases (Wilkinson and Ramsdale, 2011). Glyceraldehyde-*3*-phosphate dehydrogenase has been identified as the first *YCA1*-specific substrate degraded *en route* to H_2_O_2_-induced PCD (Silva et al., 2011), but yeast PCD mechanisms occurring both in *YCA1*-dependent and -independent manner as well as the role of other proteases in yeast PCD remain to be established (Madeo et al., 2009; Wilkinson and Ramsdale, 2011).

Both in yeast and in mammalian PCD mitochondria play a major role in final pro-survival or pro-death decision. Accordingly, the mitochondria-mediated PCD pathway in yeast resembles the mammalian intrinsic pathway, and shows remarkable complexity with respect to different proteins and pathways involved (Eisenberg et al., 2007; Pereira et al., 2008). Alterations in mitochondrial structure and function during PCD depend on a variety of specific triggers, respiratory or fermentative growth conditions, and on overall cell metabolism. First evidence for a mitochondria-dependent yeast PCD pathway was obtained in acetic acid-induced PCD (AA-PCD), with cells showing cytochrome *c* (cyt *c*) release into the cytosol and production of mitochondrial reactive oxygen species (ROS). Mitochondrial dysfunction occurs as shown by mitochondrial depolarization, and a large decrease in cyt *c* oxidase (COX) activity together with higher resistance to AA-PCD of respiratory-deficient cells, lacking either mtDNA or unable to form active cyt *c* or ATP synthase (Ludovico et al., 2002). Key regulators of mitochondrial metazoan apoptosis are the Bcl-2 family proteins which include both pro-apoptotic and anti-apoptotic members harboring multiple or single Bcl-2 homology (BH) domains (BH1-4). These proteins regulate mitochondrial outer membrane permeabilization (MOMP) followed by the release of pro-apoptotic factors including cyt *c* (Wang and Youle, 2009; Wasilewski and Scorrano, 2009). Recent discovery of a yeast BH3-only protein (Ybh3p) mediating both AA- and H_2_O_2_-induced PCD (Büttner et al., 2011) supports the hypothesis of the origin of the eukaryotic PCD systems through acquisition of several PCD effectors as a consequence of mitochondrial endosymbiosis (Koonin and Aravind, 2002). Indeed, yeast Ybh3p translocates to mitochondria inducing PCD and mitochondrial membrane depolarization through interaction with the mitochondrial phosphate carrier (Mir1p) and a core subunit of the respiratory complex III (Cor1p; Büttner et al., 2011). Thus, Ybh3p resembles mammalian Bax that can permeabilize mitochondria, whereas mammalian BH3-only proteins require Bax and Bak to release cyt *c*, suggesting that the most ancestral function of the BH3-like proteins may be to trigger changes in the IMM (Oettinghaus et al., 2011).

Whether yeast PCD does resemble and/or predate apoptotic death in multicellular organisms or is a distinct form of PCD in itself is still a matter of investigation. Indeed, it remains controversial as to whether metacaspases are distant relatives of caspases or are more closely related to other classes of proteases. Moreover even if yeast encodes a BH3-only protein as recent studies suggest, yeast homologs of Bcl-2 proteins on which BH3-only proteins act are still unknown. Notwithstanding this, the central role of mitochondria in yeast PCD underlines the importance of dissecting the PCD process in this unicellular organism.

In this review we consider the mitochondrial proteins involved in yeast PCD execution and regulation (see **Table 1**). Most of them are involved in either electron transfer along the respiratory chain and oxidative phosphorylation, or mitochondrial dynamics, or mitochondrial permeabilization and protein trafficking from mitochondria to cytosol and vice versa. These points will be dealt with separately.

**Table 1:** Yeast mitochondrial proteins involved in PCD regulation.

Gene (protein)	Mammalian homolog	PCD trigger	Role in PCD	Reference
AAC1/AAC2/AAC3 (ADP/ATP carrier isoforms)	ANT	Acetic acid, diamide, H_2_O_2_	MOMP	Pereira et al. (2007)
AIF1 (apoptosis-inducing factor)	AIF	Acetic acid, bostrycin, H_2_O_2_	Pro-apoptotic released factor translocating to the nucleus	Wissing et al. (2004), Xu et al. (2010)
ATP10 (ATP synthase assembly factor)	ATP synthase	Acetic acid	Pro-apoptotic factor	Ludovico et al. (2002)
CIT1 (citrate synthase)	CS	Aging, heat	GSH biosynthesis, antioxidant activity	Lee et al. (2007)
COR1 (complex III core subunit)	QCR1	Acetic acid +Ybh3 overexpression	ETC,YBH3 interaction	Büttner et al. (2011)
CYC1/CYC7 (cytochrome c isoforms 1, 2)	Cytochrome c	Acetic acid, amiodarone/α-factor, ASF1/CIA1 deletion, aspirin, cdc48^S565G^, Bax heterologous expression, H_2_O_2_, hyperosmotic stress, salt stress	Pro-apoptotic released factor, ETC electron donor, ROS scavenger	Manon et al. (1997), Yamaki et al. (2001), Ludovico et al. (2002), Pozniakovsky et al. (2005), Silva et al. (2005), Braun et al. (2006), Pereira et al. (2007), Giannattasio et al. (2008), Sapienza et al. (2008), Gao et al. (2011)
CYC3 (cytochrome c heme lyase)	CCHL	Acetic acid, amiodarone, hyperosmotic stress	Cyt *c* holoenzyme formation	Ludovico et al. (2002), Pozniakovsky et al. (2005), Silva et al. (2005)
FIS1 (mitochondrial fission protein)	hFIS	Acetic acid, BAR0329, ethanol, heat shock, H_2_O_2_	Mitochondrial dynamics	Fannjiang et al. (2004), Kitagaki et al. (2007), Bink et al. (2010)
L14-A (mitochondrial 60S ribosomal protein)	–	Grapefruit seed extract	Unknown	Cao et al. (2012)
MIR1 (mitochondrial phosphate carrier)	PHC	Acetic acid +Ybh3 overexpression	Energetic metabolism,YBH3 interaction	Büttner et al. (2011)
NDI1 (internal NADH dehydrogenase)	AMID	NDI1 overexpression	ROS production	Li et al. (2006)
NUC1 (mitochondrial nuclease)	Endo G	Acetic acid, amiodarone, ethanol, H_2_O_2_	Pro-apoptotic released factor translocating to the nucleus	Büttner et al. (2007), Kitagaki et al. (2007)
POR1 (porin)	VDAC	Acetic acid, H_2_O_2_,diamide	Anti-apoptotic factor	Pereira et al. (2007)
RSM23 (mitochondrial 40S ribosomal protein)	hDAP-3	YCA1 overexpression	Pro-apoptotic factor	Madeo et al. (2002)
TIM18 (translocase of the inner mitochondrial membrane)	–	Arsenite	MOMP	Du et al. (2007)
YME1 (catalytic subunit of i-AAA protease complex)	–	Heterologous expression of Bax	Complex IV degradation	Manon et al. (2001)
YSP1 (yeast suicide protein 1)	–	α-Factor, amiodarone	Mitochondrial dynamics	Pozniakovsky et al. (2005)
YSP2 (yeast suicide protein 2)	–	Acetic acid, amiodarone	Mitochondrial dynamics	Sokolov et al. (2006)

* The S. cerevisiae mitochondrial proteins reported in this table have been implicated in PCD induced by different triggers through biochemical and/or genetic studies.ANT, adenine nucleotide translocator; MOMP, mitochondrial outer membrane permeabilization; CS, citrate synthase; GSH, glutathione; QCR1, ubiquinol–cytochrome c reductase core protein;YBH3, yeast BH3-only; ETC, electron transport chain; CCHL, cyt c heme lyase; hFIS, human homolog of Fis1p; BAR0329, 4-{[3-(4-chlorobenzyl)- 2-methoxyquinolin-6-yl]methyl}piperazine-1-carboximidamide; PHC, phosphate carrier; AMID, apoptosis-inducing factor-homologous mitochondrion-associated inducer of death; Endo G, endonuclease G; VDAC, voltage-dependent anion channel; hDAP-3, human death associated protein.*

## ELECTRON TRANSFER ALONG THE RESPIRATORY CHAIN AND OXIDATIVE PHOSPHORYLATION

Yeast internal NADH dehydrogenase (NDI1) is the homolog of metazoan AMID, the apoptosis-inducing factor (AIF)-homologous mitochondrion-associated inducer of death. Ndi1p overexpression can cause PCD, probably due to ROS production in mitochondria, only when cells are grown in glucose-rich media. However this occurs in yeast cells lacking mitochondrial superoxide dismutase both during fermentative and respiring growth (Li et al., 2006). Yme1p is a mitochondrial AAA-type protease involved in the coordinated assembly of COX. Yme1p activation results in a decrease of COX level *en route* to Bax-induced cell death; however since under fermentative conditions, when COX activity is strongly repressed, *YME1* deletion slightly delays Bax-induced cell death, some other unidentified Yme1p substrate could also play a role in this process (Manon et al., 2001). Analysis of the effect of oxidative phosphorylation inhibitors on yeast PCD has shown conflicting results depending on the PCD trigger. Although AA-PCD is insensitive to antimycin or oligomycin, myxothiazol and cyanide prevented amiodarone/α-factor-induced PCD (Ludovico et al., 2002; Pozniakovsky et al., 2005; Guaragnella et al., 2011b). Yeast cells grown in the presence of both antimycin and oligomycin and subsequently treated with acetic acid in the presence of both these compounds displayed a higher sensitivity to AA-PCD (Pereira et al., 2007). Yet, fully assembled and functional F_0_F_1_-ATPase and cyt *c* are required for Bax-induced PCD and AA-PCD to occur (Matsuyama et al., 1998; Ludovico et al., 2002; Guaragnella et al., 2011a).

Thus, complexes participating in oxidative phosphorylation have key roles in yeast PCD different from electron transport and ATP synthesis, likely ROS production. Interestingly, deletion of mitochondrial citrate synthase (*CIT1*) results in higher sensitivity to oxidative stress and PCD induction, due to impairment of reduced glutathione (GSH) biosynthesis (Lee et al., 2007), suggesting that other metabolic pathways are also involved in oxidative stress.

## MITOCHONDRIAL DYNAMICS

Extensive mitochondrial fragmentation is recognized as a general feature in yeast PCD. Fis1p, Dnm1p, and Mdv1p/Net2p, which constitutes the machinery responsible for mitochondrial fission in healthy cells (Fannjiang et al., 2004), are involved in mitochondrial fragmentation/degradation and cell death induced by different stimuli (Fannjiang et al., 2004; Kitagaki et al., 2007; Bink et al., 2010). Indeed, *DNM1* gene deletion extends life span by increasing cellular resistance to PCD induction (Scheckhuber et al., 2007). In distinction from its pro-apoptotic function in mammals, yeast Fis1p is a mitochondrial protein which inhibits *DNM1*-mediated cell death by inhibiting the fission function of Dnm1p, differently from its role in mitochondrial fission during normal growth (Fannjiang et al., 2004). This inhibitory function of Fis1p can be functionally replaced by human Bcl-2 and Bcl-x_L_, supporting the idea that Fis1p is a functional homolog of anti-apoptotic Bcl-2 family proteins (Cheng et al., 2008a) and, together with Ybh3p (Büttner et al., 2011), is a component of an ancestral mitochondrial PCD pathway. The pro-survival role of *FIS1* was confirmed in studies using different apoptotic triggers, such as virus-encoded toxin, ethanol, and fungicidal derivative BAR0329 (Ivanovska and Hardwick, 2005; Kitagaki et al., 2007; Bink et al., 2010). However, *FIS1* may have an additional long-term survival function which appears to be independent of *DNM1* and *MDV1*. Indeed, *FIS1* deletion results in acquisition of a secondary mutation in the stress-response gene *WHI2* that confers sensitivity to cell death (Cheng et al., 2008b).

Genetic screens have revealed the existence of two novel genes, named yeast suicide protein 1 (*YSP1*) and yeast suicide protein 2 (*YSP2*), required for mitochondrial fragmentation *en route* to amiodarone-induced PCD (Pozniakovsky et al., 2005; Sokolov et al., 2006). It has been proposed that Ysp2p acts downstream of ROS production due to intracellular acidification, following AA-PCD induction (Sokolov et al., 2006). No homologous genes have been found in higher organisms.

## MITOCHONDRIAL PERMEABILITY AND PROTEIN TRAFFICKING FROM MITOCHONDRIA TO CYTOSOL AND VICE VERSA

As in mammals, the release of pro-apoptotic mitochondrial proteins occurs *en route* to yeast PCD. Cyt *c* was the first mitochondrial protein shown to have an apoptotic function different from its role as an electron carrier in the respiratory chain. Cyt *c* release from mitochondria occurs commonly in yeast PCD both in response to a variety of stimuli, including acetic acid (Ludovico et al., 2002; Giannattasio et al., 2008), amiodarone/α-factor (Pozniakovsky et al., 2005), H_2_O_2_ (Pereira et al., 2007), aspirin (Sapienza et al., 2008), salt stress (Gao et al., 2011), and as a result of heterologous expression of the mammalian BAX (Manon et al., 1997). Cyt *c* release was also observed in yeast strains lacking the histone chaperone *ASF1/CIA1* (Yamaki et al., 2001) and with a mutation in *CDC48* (cdc48^S565G^; Braun et al., 2006). Deletion of cyt *c* isoforms or heme lyase, necessary for cyt *c* maturation, inhibits yeast PCD triggered by different stimuli (Ludovico et al., 2002; Severin and Hyman, 2002; Pozniakovsky et al., 2005; Silva et al., 2005; Yang et al., 2008; Gao et al., 2011), except ethanol (Kitagaki et al., 2007).

Mammalian AIF is a FAD-containing oxidoreductase localized in the mitochondrial intermembrane space whose specific enzymatic activity remains unknown (Sevrioukova, 2011). AIF is a caspase-independent death effector and also plays a vital mitochondrial role in healthy cells (Hangen et al., 2010). Similarly to AIF, the yeast homolog Aif1p translocates to the nucleus in response to apoptotic stimuli (Wissing et al., 2004). *AIF1* disruption rescues yeast cells from oxygen stress and delays age-induced PCD. Conversely, overexpression of *AIF1* strongly stimulates H_2_O_2_-induced PCD; this effect is attenuated by disruption of *YCA1*. Contrarily, *AIF1*-dependent bostrycin-induced cell death was shown to be independent of *YCA1* (Xu et al., 2010).

Nuc1p is the yeast homolog of metazoan endonuclease G (EndoG), a mitochondrial protein with DNase/RNase activity involved in apoptotic DNA degradation (Li et al., 2001). Overexpression of Nuc1p promotes yeast PCD. Nuc1p-mediated PCD is shown to be *AIF1*- and *YCA1*-independent, which favors the existence of multiple, redundant pathways regulating cell death. Nuc1p translocates from mitochondria to the nucleus upon death induction. Nuc1p-dependent death depends on its interaction with *AAC2*, as well as with histone H2B and *KAP123*, coding for karyopherin involved in nuclear import, indicating that the pro-death role of Nuc1p requires nuclear import and chromatin association (Büttner et al., 2007). When mitochondrial respiration is increased *NUC1* deletion inhibits apoptotic death, whereas under respiration repressing conditions, *NUC1* deletion sensitizes yeast cells to non-apoptotic death, this showing a dual, pro-life and pro-death role for *NUC1* (Büttner et al., 2007; Kitagaki et al., 2007).

The yeast genome also harbors a gene, called *NMA111*, homologous to vertebrate HtrA2/Omi mitochondrial serine protease, which mediates apoptosis once released to the cytosol where it can antagonize the inhibitor of apoptosis protein XIAP (Vande Walle et al., 2008). Differently from HtrA2/Omi, yeast Nma111p is a nuclear protein that, under cellular stress conditions such as H_2_O_2_-induced PCD, tends to aggregate inside the nucleus without its expression level being upregulated, suggesting that aggregation of Nma111p is correlated to its death-mediating character (Fahrenkrog et al., 2004).

Mitochondrial protein release and MOMP are crucial events in yeast PCD. Certain mitochondrial proteins possibly involved in MOMP *en route* to yeast PCD have been identified. Yeast possesses the homologous genes of the putative core components of mammalian permeability transition pore, ADP/ATP carrier proteins (*AAC1,2,3*), yeast voltage-dependent anion channel (*POR1*), and a mitochondrial cyclophilin (*CPR3*). While Por1p was proposed to have a pro-survival role and Cpr3p had no effect on yeast PCD, only deletion of AAC proteins was shown to protect cells from AA- but not H_2_O_2_-induced PCD, and to inhibit cyt *c* release (Pereira et al., 2007). In addition, the AAC proteins and the vacuolar protease Pep4p have been shown to have a role in mitochondrial degradation *en route* to AA-PCD; Pep4p is released from the vacuole upon AA-PCD induction, suggesting a vacuole-mitochondrial cross-talk during yeast PCD (Pereira et al., 2010).

The mitochondrial inner membrane translocase, Tim18, was shown to be involved in arsenic-induced yeast cell death (Du et al., 2007), this raising a question about the possible involvement of this translocase in MOMP. Tim18 is part of the Tim54–Tim22 complex, Tim22 being a mitochondrial receptor for the pro-apoptotic protein Bax (Kovermann et al., 2002).

Two other proteins, Mmi1p and Mcd1p, have been shown to translocate to mitochondria *en route* to yeast PCD. The former functionally links microtubules and mitochondria (Rinnerthaler et al., 2006). The latter causes the decrease of mitochondrial membrane potential amplifying PCD in a cyt *c*-dependent manner (Yang et al., 2008).

## A CASE STUDY: THE ROLE OF CYTOCHROME *c* IN YEAST PCD

Although cyt *c* release occurs *en route* to yeast PCD, so far in *S. cerevisiae* there is no evidence of the existence of a functional homolog of the apoptosome (Huttemann et al., 2011). Accordingly, yeast cyt *c* is unable to activate caspases in cytosolic extracts from metazoan cells (Kluck et al., 2000; Bender et al., 2012). Thus, some questions need to be answered: which event/s triggers cyt *c* release? Is cyt *c* released from damaged mitochondria? What is the role of the released cyt *c* *en route* to PCD, and is it strictly required for PCD to occur? In this regard, the definition of the sequence of events leading to the death cascade turns out to be useful.

**FIGURE 1:**
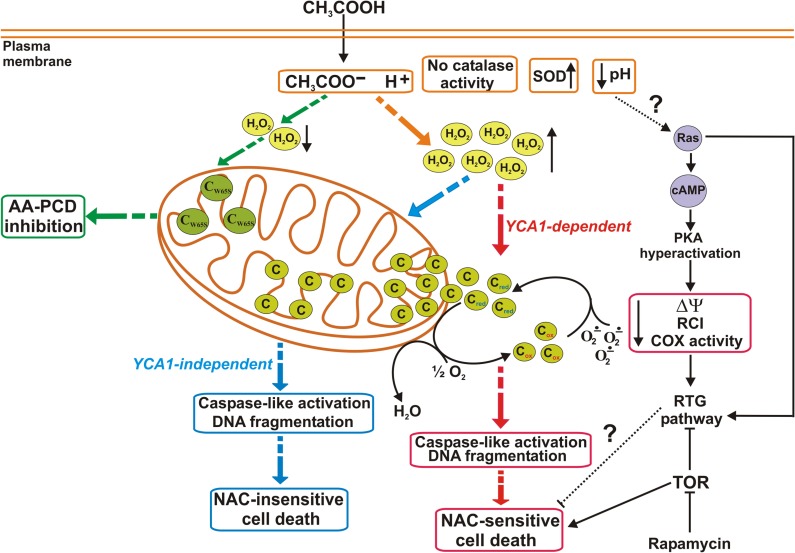
**Cytochrome *c* and mitochondrial dysfunction in AA-PCD pathways.** At extracellular acidic pH values acetic acid enters yeast cells and dissociates into acetate and protons causing intracellular acidification. In NAC-sensitive AA-PCD (red dashed arrows) hydrogen peroxide (H_2_O_2_) accumulates early, superoxide dismutase (SOD) activity increases, while catalase activity is undetectable; cyt *c* is released to the cytosol in a YCA1-dependent manner as a functional protein, acting as an electron donor (c_red_) to the electron transport chain and as a superoxide anion (O_2_^-^^.^) scavenger; in a late phase, mitochondrial functions progressively decline, as revealed by a decrease in mitochondrial membrane potential (ΔΨ), respiratory control index (RCI), and cyt *c* oxidase (COX) activity. Caspase-like activity increases and DNA fragmentation occurs. The NAC-insensitive (blue dashed arrows) AA-PCD takes place in a YCA1-independent manner without cyt *c* release, yet caspase-like activation and DNA fragmentation occur in a late phase. In cells expressing a catalytically inactive form of iso-1-cyt *c* (C_W65S_; green dashed arrows), no release of mutant cyt *c* occurs with inhibition of AA-PCD, and there is a decrease in H_2_O_2_ production. Possible involvement of certain signaling pathways in the interplay between PCD and cell adaptation is also shown: intracellular acidification caused by AA-PCD induction may stimulate RAS–cAMP–PKA signaling pathway, causing mitochondrial dysfunction, which can activate retrograde (RTG) pathway. The RTG pathway is positively and negatively regulated by Ras and TOR pathways, respectively. The TOR pathway is found at the crossroad of AA-PCD and RTG signaling, which may play a role in AA-PCD resistance.

After the discovery of the occurrence of AA-PCD in yeast (Ludovico et al., 2001, 2002), in a series of papers a detailed time course of certain events was investigated (Giannattasio et al., 2005, 2008; Guaragnella et al., 2006, 2007, 2008, 2010a; Ribeiro et al., 2006; Pereira et al., 2007). These events can be classified as pre- and post-cyt *c* release (**Figure 1**). Loss of cell viability is complete after 200 min of acetic acid treatment with accumulation of cells with fragmented nuclear DNA. The earliest event (15 min) following acetic acid challenge is ROS production, with a different role for H_2_O_2_ and superoxide anion, whose levels are modulated by catalase and superoxide dismutase. *En route* to death cyt *c* starts to be released at 60 min from coupled and intact mitochondria; maximum release is reached at 150 min. Later on cyt *c* is degraded, possibly by yet unidentified proteases. The latest event of AA-PCD is caspase-like activation occurring at 200 min from death induction. Mitochondria are functionally implicated in this death scenario. In fact, up to 150 min released cyt *c* can act both as an electron donor as well as a ROS scavenger. However, *en route* to death a progressive impairment of mitochondrial functions, evidenced by a decrease of the respiratory control index, a collapse of the mitochondrial membrane potential, a decrease in COX activity and in cytochromes *a* + *a*_3_ levels, have been observed.

The AA-PCD time course clearly shows that ROS accumulation and caspase-like activation occur upstream and downstream of cyt *c* release, respectively. Functional genomics and biochemical studies on knock-out cells lacking *YCA1* and/or the genes encoding the two yeast cyt *c* isoforms allowed the elucidation of causal relationships among ROS levels, cyt *c* release and caspase-like activation and two separate pathways activated by acetic acid have been identified. Particularly, it has been found that ROS and *YCA1* are required for cyt *c* release, since both prevention of ROS production by the antioxidant *N*-acetyl cysteine (NAC) and *YCA1* disruption result in the inhibition of cyt *c* release (Guaragnella et al., 2010a,b). How *YCA1* is related to cyt *c* release remains to be elucidated. Nevertheless, a recent report suggests that *YCA1* has a role in mitochondrial respiratory functions (Lefevre et al., 2012). Interestingly, AA-PCD still occurs, although with a lower death rate compared to wild type cells, without cyt *c* release in ADP/ATP carrier as well as *YCA1* and/or cyt *c* knock-out cells (Pereira et al., 2007; Guaragnella et al., 2010b). This confirms on one hand that *YCA1* and cyt *c* act as pro-apoptotic proteins in yeast AA-PCD, but on the other hand that they are dispensable for PCD occurrence, showing the existence of *YCA1*/cyt *c*-independent AA-PCD pathway (**Figure 1**). In this pathway ROS accumulate early, caspase-like activity increase, and DNA fragmentation occurs. Importantly, *YCA1*/cyt *c*-independent AA-PCD is insensitive to NAC. This evidence suggests that cyt *c* still present in mitochondria might play a role in AA-PCD. Recent studies performed on yeast cells expressing a stable but catalytically inactive iso-1-cyt *c* (W65Scyc1) unable to reduce COX have shown inhibition of AA-PCD, with a decrease of ROS production, no cyt *c* release, this being independent of electron flow impairment, and an increase in caspase-like activation (**Figure 1**). Thus, cyt *c* release does not depend on cyt *c* function as an electron carrier and when still associated to the mitochondrial membrane, cyt *c* in its reduced form has a role in AA-PCD by regulating ROS production and caspase-like activity (Guaragnella et al., 2010a,b, 2011b). Regulation of ROS production by mitochondrial cyt *c* during AA-PCD may be exerted either directly by the cyt *c* peroxidase system able to scavenge both superoxide anion and H_2_O_2_ (Korshunov et al., 1999) or by a change in cyt *c*–cardiolipin interaction or inefficient cardiolipin peroxidation by ROS (Kagan et al., 2005; Bayir et al., 2006; Sinibaldi et al., 2010; Huttemann et al., 2011). These issues require further investigations.

## CONCLUSIONS AND PERSPECTIVES

In the light of results emerging from research into yeast PCD we feel that there is consensus that the response to any stimulus leading to PCD depends on the intrinsic status of the cells, for instance the growth phase or the metabolic and environmental conditions. Paradigmatic of this is that in response to acetic acid in cells with increased mitochondrial respiration yeast activates a Nuc1p-dependent PCD pathway (Büttner et al., 2007), whereas in stationary growth phase yeast is less sensitive to acetic acid (Ludovico et al., 2002), and after acid stress adaptation it is highly resistant to AA-PCD induction (Giannattasio et al., 2005; Ždralević et al., 2012).

Although a number of mitochondrial proteins participating in yeast PCD have been identified, how they work *en route* to PCD remains to be fully established. Further aspects also need to be investigated, including the fact that mitochondria are important organelles in the cross-talk between death- and life-promoting signaling pathways. Indeed, RAS–cAMP–PKA (Longo, 2003; Roosen et al., 2005; Gourlay et al., 2006; Leadsham and Gourlay, 2010), target of rapamycin (TOR) kinase (Almeida et al., 2009) and retrograde (Jazwinsky, 2003; Liu and Butow, 2006) signaling pathways have been shown to control yeast cell PCD and aging through mitochondrial function regulation (**Figure 1**).

Mitochondrial dysfunction and the mode of cell response to it underlie different pathological conditions such as neurodegeneration. Alterations in mitochondrial functions have long been observed also in cancer cells and targeting mitochondria as an anti-cancer therapeutic strategy has gained momentum recently (Gogvadze et al., 2009). Since yeast shares with cancer cells the metabolic features identified as the underlying causes of the Warburg effect (Ruckenstuhl et al., 2009; Diaz-Ruiz et al., 2010), it is a suitable model organism to identify cell compounds responsible for tumorigenesis for development of targeted cancer drugs.

## Conflict of Interest Statement

The authors declare that the research was conducted in the absence of any commercial or financial relationships that could be construed as a potential conflict of interest.
